# Supplementary material for: Chemical Profile and Screening of Bioactive Metabolites of Rindera graeca (A. DC.) Bois. & Heldr. (Boraginaceae) In Vitro Cultures
Source: Plants (Basel). 2021 Apr 21;10(5):834. doi: 10.3390/plants10050834 (PMC8143363; doi:10.3390/plants10050834)
Supplement: Supplementary file 1 [file plants-10-00834-s001.zip › plants-1192124-supplementary.pdf]

Supplementary

Chemical Profile and Screening of Bioactive Metabolites of *Rindera graeca* (A. DC.) Bois. & Heldr. (Boraginaceae) In Vitro cultures

Konstantia Graikou [1], Harilaos Damianakos [1], Christos Ganos [1], Katarzyna Sykłowska-Baranek [2], Małgorzata Jeziorek [2], Agnieszka Pietrosiuk [2], Christos Roussakis [3] and Ioanna Chinou [1,*]

[1] Laboratory of Pharmacognosy and Chemistry of Natural Products, Department of Pharmacy, National & Kapodistrian University of Athens, 15771 Zografou, Athens, Greece; kgraikou@pharm.uoa.gr (K.G.); harisdam@pharm.uoa.gr (H.D.); chris50ganos@hotmail.com (C.G.)

[2] Department of Pharmaceutical Biology and Medicinal Plant Biotechnology, Faculty of Pharmacy, Medical University of Warsaw, 1 Banacha, 02-097 Warsaw, Poland; kasiasb@farm.amwaw.edu.pl (K.S.-B.); gosiajeziorek@op.pl (M.J.); agnieszka.pietrosiuk@wum.edu.pl (A.P.)

[3] IICi MED/EA 1155- Department Cancer du Poumon et Cbles Moleculaires, UFR Sciences Pharmaceutiques- 9 rue Bias, CEDEX 1, 44035 Nantes, France; christos.roussakis@univ-nantes.fr

* Correspondence: ichinou@pharm.uoa.gr

Rosmarinic acid [1, 2]

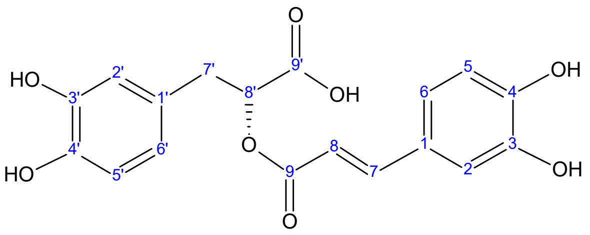

^{1}H-NMR

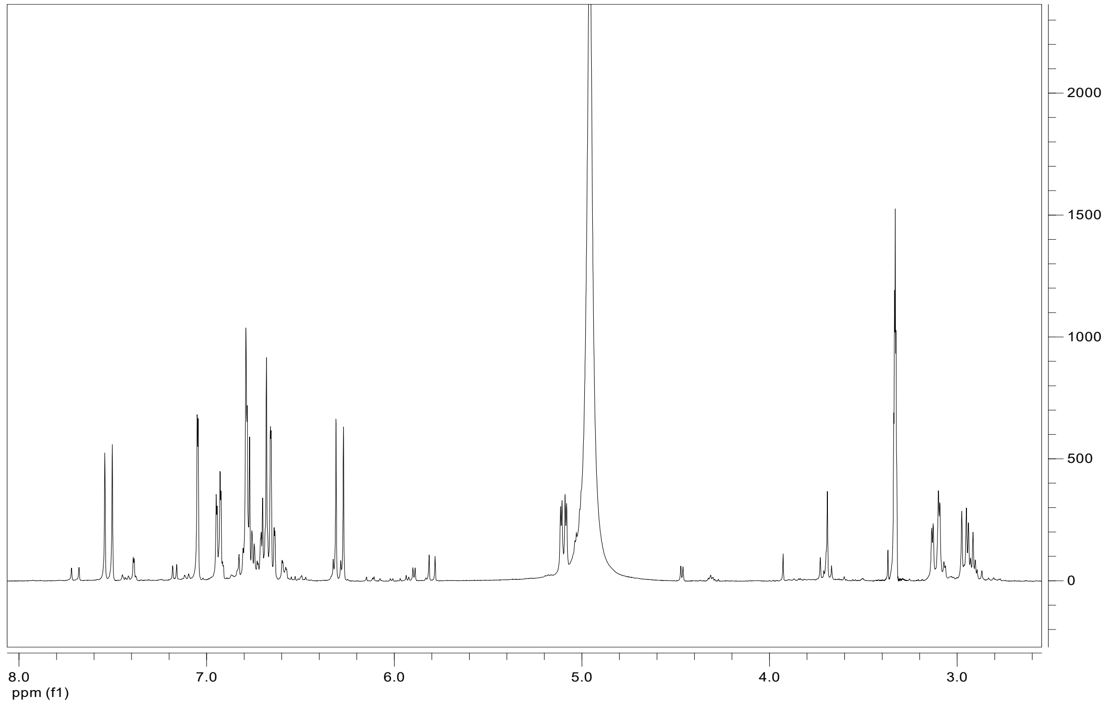

position	[1]H, δ (ppm)	multiplicity	J (Hz)
1			
2	7.03	d	2.0
5	6.77	d	8.0
6	6.92	d	8.0
7	7.50	d	16.0
8	6.27	d	16.0
2′	6.76	s	
5′	6.67	d	8.0
6′	6.63	d	8.0
7′	2.93	dd	9.4/14,6
	3.10	dd	2.8/14.6
8′	5.08	dd	2.8/9.6

Rabdosiin disodium salt [1]

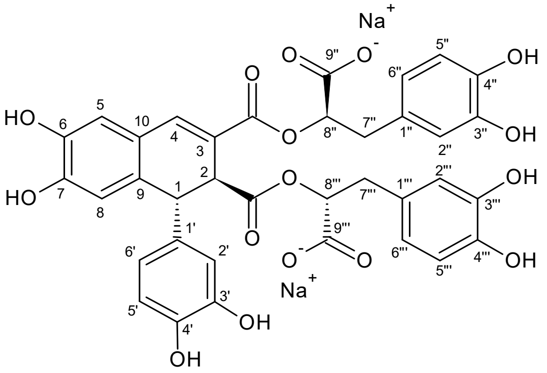

^{1}H-NMR

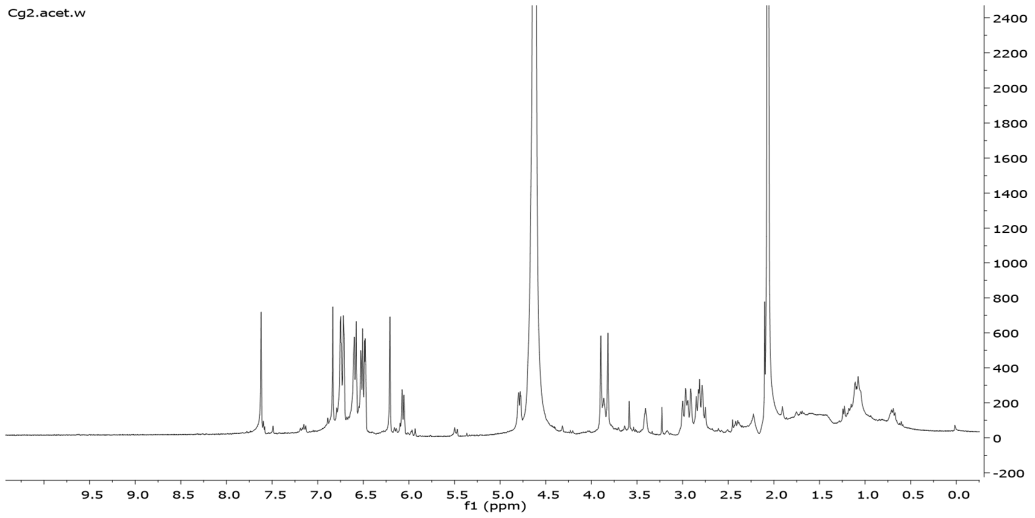

position	[1]H, δ (ppm)	multiplicity	J (Hz)
1	3.88	brs	
2	3.80	brs	
4	7.60	s	
5	6.82	s	
8	6.46	s	
2'	6.19	s	
5'	6.48	d	8.1
6'	6.04	d	8.1
2''	6.70	s	
5''	6.50	d	8.1
6''	6.57	d	8.1
7''	2.80 / 2.93	m	
8''	4.77	dd	8.9, 2.8
2'''	6.73	s	
5'''	6.71	d	8.1
6'''	6.57	d	8.1
7'''	2.80 / 2.93	m	
8'''	4.66*		

*data from HMBC.

Rinderol [3]

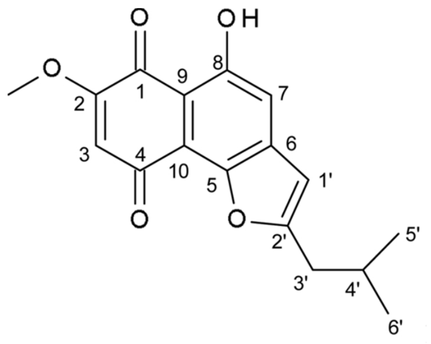

^{1}H-NMR

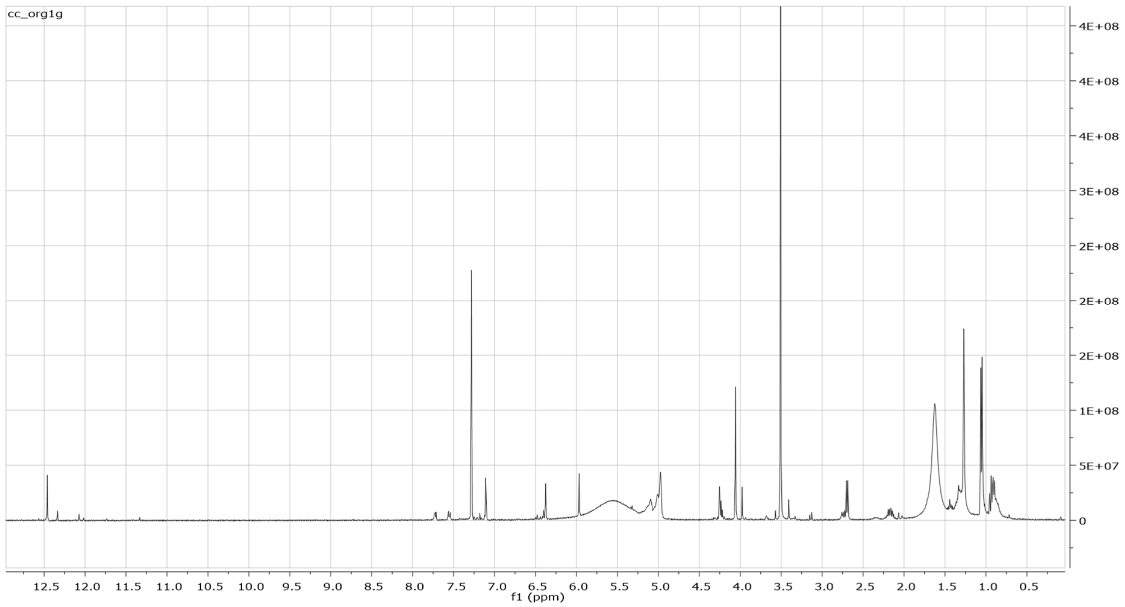

position	[1]H, δ (ppm)	multiplicity	J (Hz)
3	5.94	s	
7	7.08	s	
1′	6.35	s	
3′	2.67	d	5.2
4′	2.14	m	
5′	1.03	d	5.2
6′	1.03	d	5.2
8-OH	12.43	s	
CH_3O	4.03	s	

Quercetin 3-rutinoside-7-rhamnoside [2]

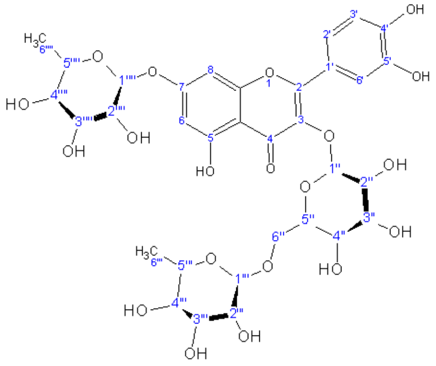

^{1}H-NMR

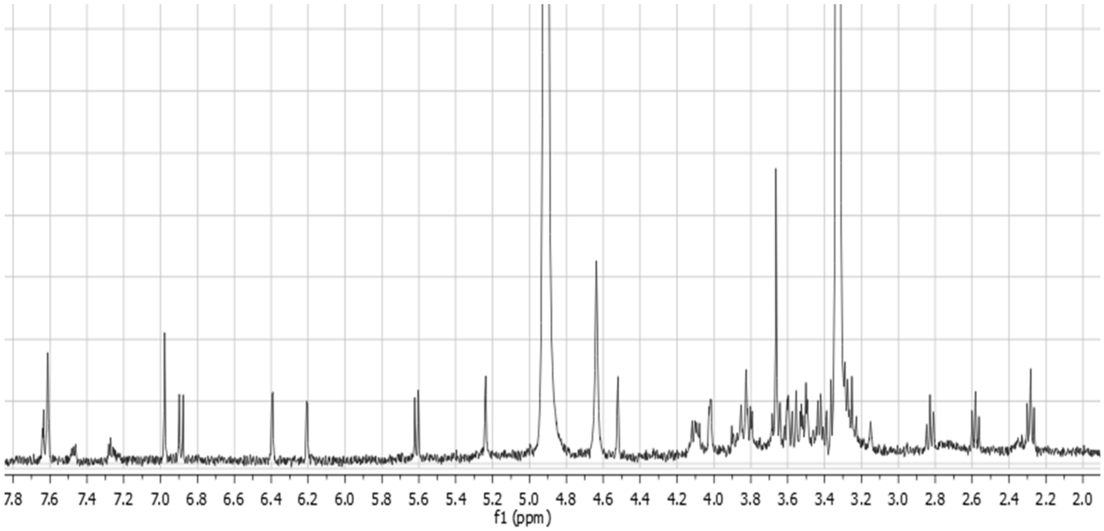

position	^{1}H, δ (ppm)	multiplicity	J (Hz)
Quercetin			
6	6.21	d	2.0
8	6.39	d	2.0
2′	7.64	d	2.0
5′	6.89	d	8.5
6′	7.63	dd	2.0 / 8.0
3-glucoside			
1′′	5.60	d	7.2
2′′ - 5′′	3.26 – 2.40	m	
6’’- rhamnoside			
1’’’	4.52	brs	
2’’’ – 5’’’	3.27 – 3.62	m	
6’’’	1.10	d	6.3
7 - rhamnoside			
6’’’’	1.02	d	6.3
1’’’’	5.22	brs	

Echinatine-N-oxide [4]

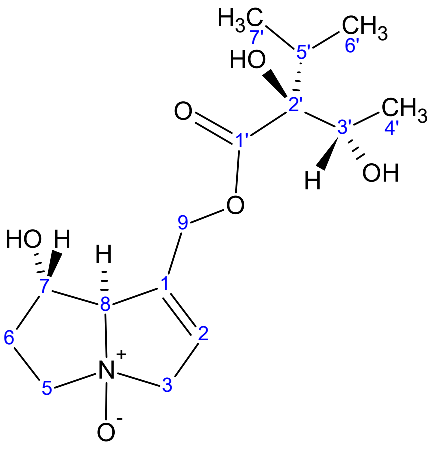

¹H-NMR

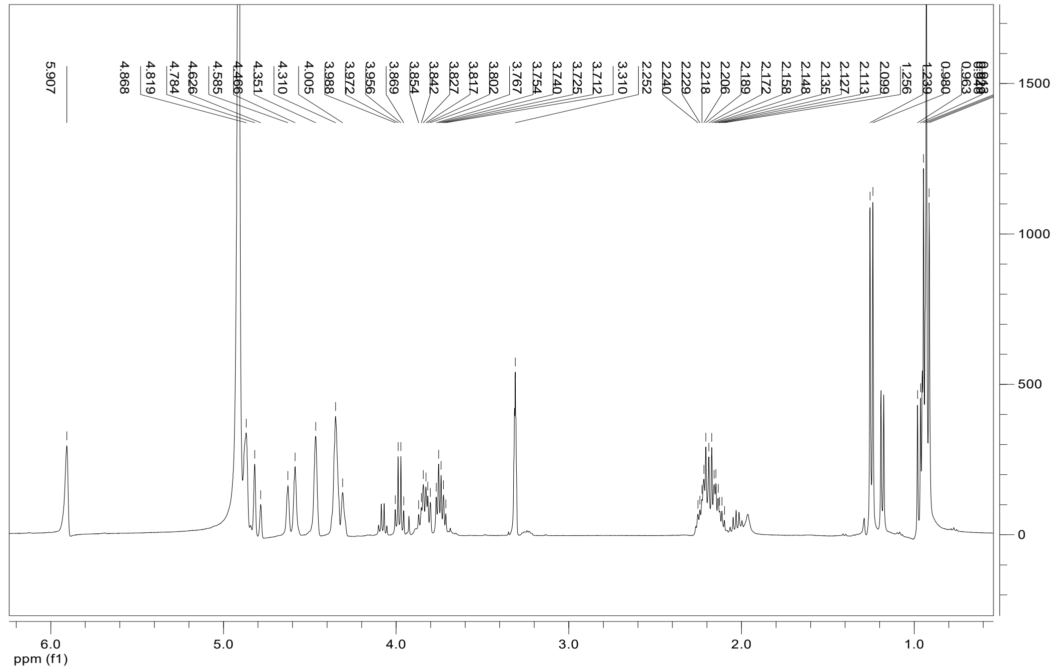

^{13}C-NMR

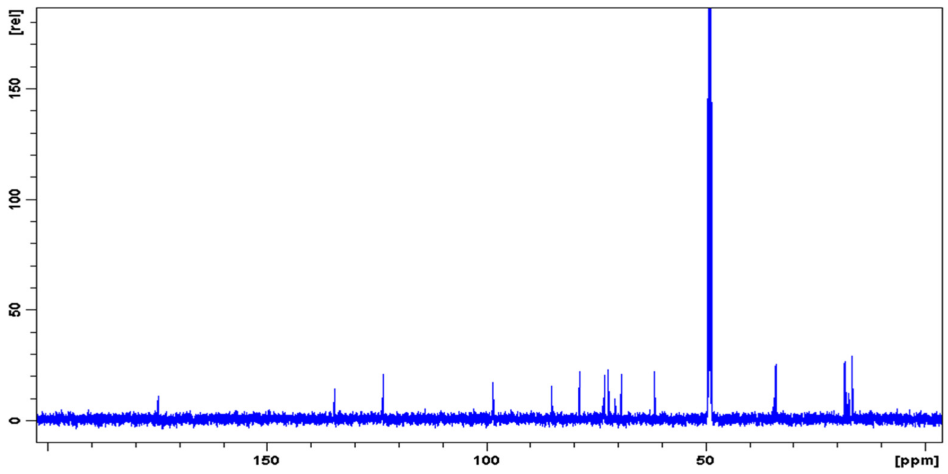

position	^{13}C, δ (ppm)	^{1}H, δ (ppm), J (Hz)
1′	174.8	
1	134.5	
2	123.4	5.91 (1H, *br s*)
8	98.4	4.46 (1H, *br s*)
2′	85.1	
3	78.7	4.33 (1H, *d*, ^{2}J=16.4)/4.60 (1H, *d*, ^{2}J=16.4)
7	73.1	4.35 (1H, *br s*)
3′	72.1	3.96 (1H, *q*, ^{3}J=6.8)
5	69.2	3.74 (1H, *ddd*, ^{2}J=10.0,^{3}J=6.0, 5.2)/ 3.83 (1H, *ddd*, ^{2}J=10.0,^{3}J=6.0, 5.2)
9	61.6	4.79 (1H, *d*, ^{2}J=14.0)/4.88 (1H, *d*, ^{2}J=14.0)
5′	34.2	2.19 (1H, *m*, ^{3}J=6.8)
6	33.9	2.14 (1H, *m*)/2.21(1H, *m*)
4′	18.3	1.24 (3H, *d*, ^{3}J=6.8)
6′,7′	18.1/16.5	0.92/0.94 (3H, *d*, ^{3}J=6.8)

Rinderine –N-oxide [4]

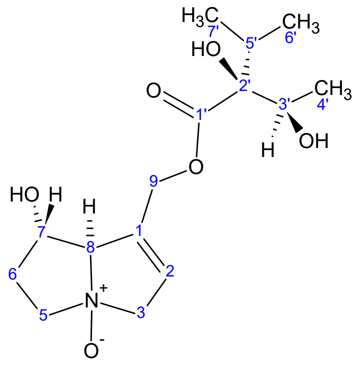

^{1}H-NMR

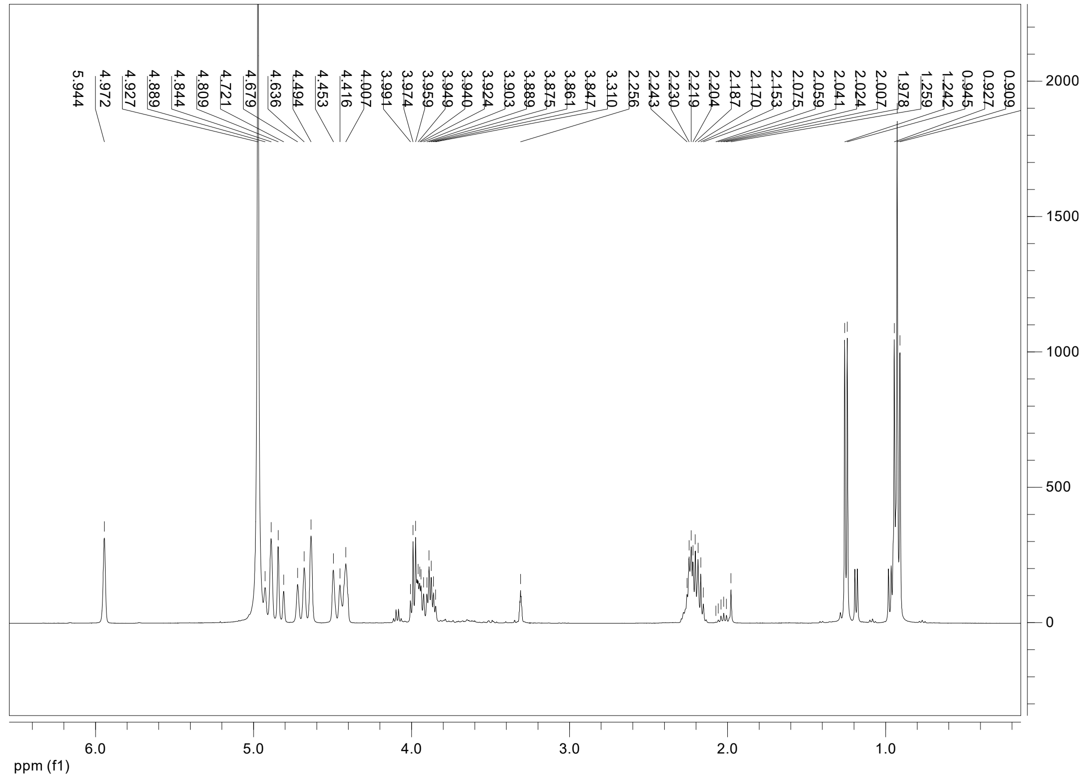

^{13}C-NMR

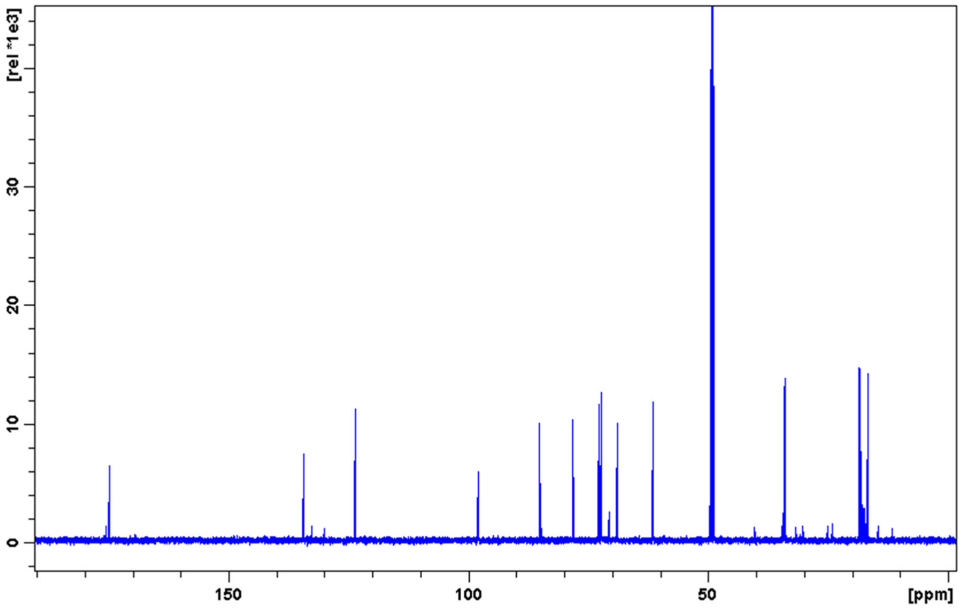

position	^{13}C, δ (ppm)	^{1}H, δ (ppm), J (Hz)
1′	174.7	
1	134.2	
2	123.4	5.94 (1H, *br s*)
8	97.8	4.64 (1H, *br s*)
2′	85.1	
3	78.0	4.47 (1H, *d*, ^{2}J=16.4)/4.70 (1H, *d*, ^{2}J=16.4)
7	72.7	4.42 (1H, *br m*)
3′	72.2	3.98 (1H, *q*, ^{3}J=6.8)
5	68.9	3.87 (1H, *m*)/3.96 (1H, *m*)
9	61.4	4.82 (1H, *d*, ^{2}J=14.0)/4.91 (1H, *d*, ^{2}J=14.0)
5′	33.9	2.20 (1H, *sept*, ^{3}J=7.0)
6	34.1	2.23 (2H, *m*)
4′	18.3	1.25 (3H, *d*, ^{3}J=6.8)
6′,7′	18.1/16.5	0.92/0.94 (3H, *d*, ^{3}J=7.0)

HPLC spectra of PA/PANOs

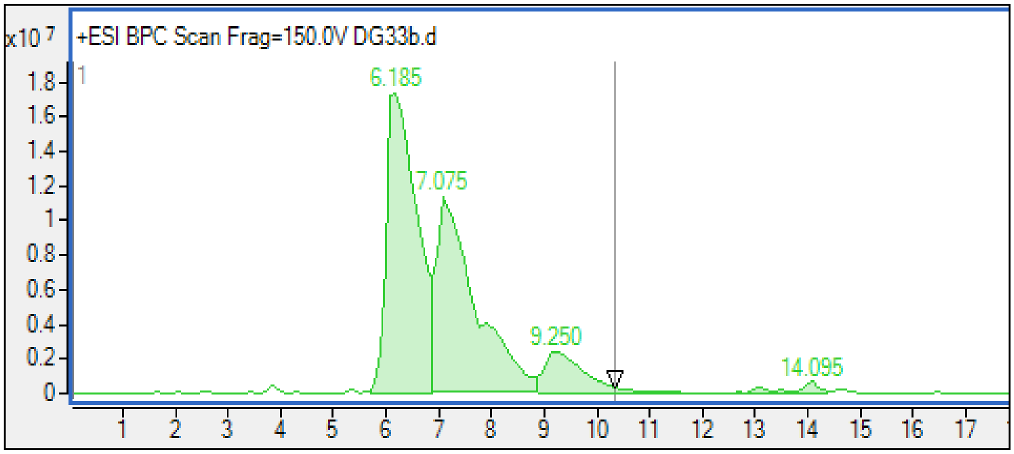

Echinatine-N-oxide [2]

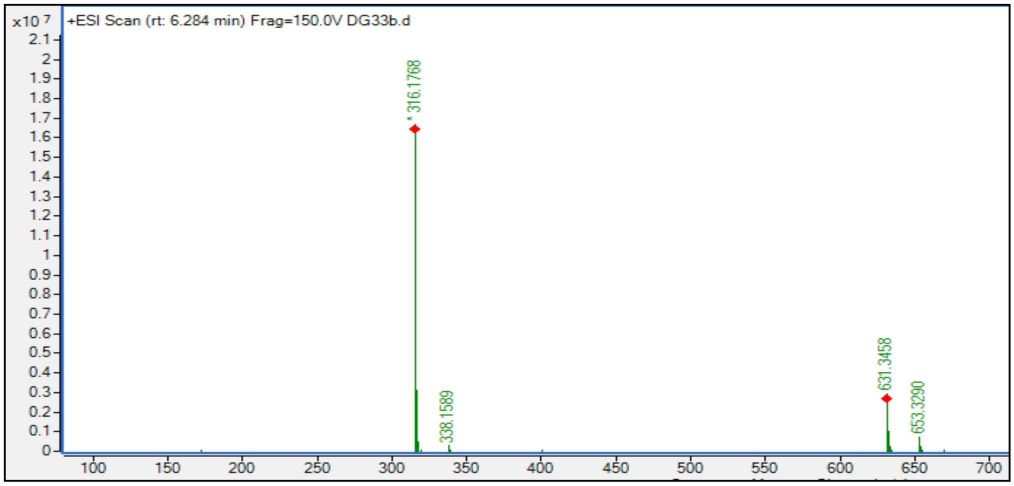

Rinderine –N-oxide [2]

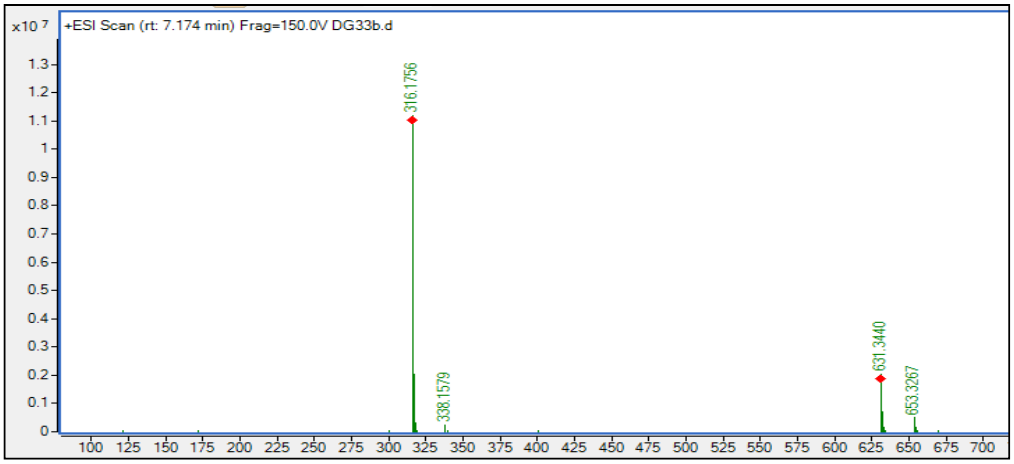

Echinatine [2]

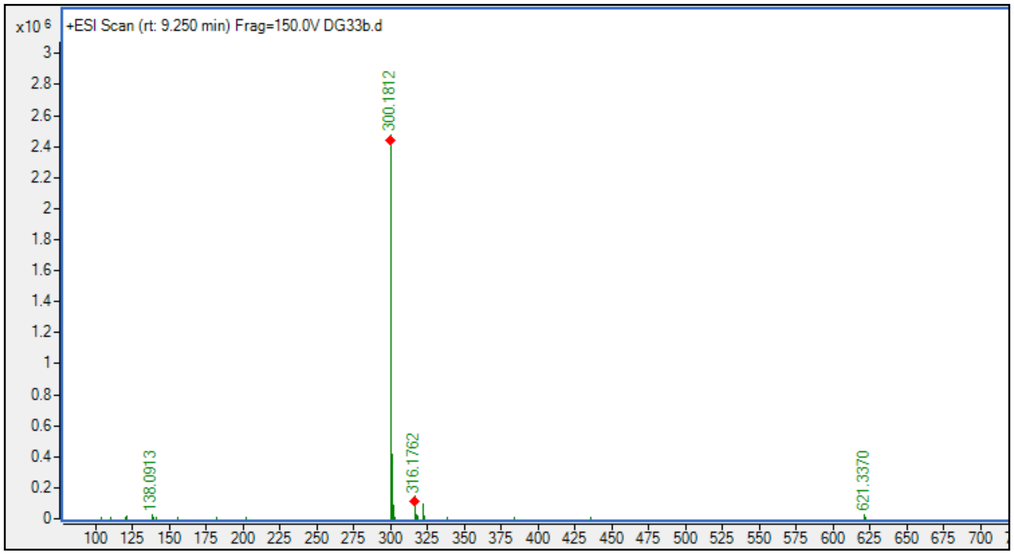

References

1. Tufa, T., Damianakos, H., Zengin, G., Graikou, K., Chinou, I. Antioxidant and enzyme inhibitory activities of disodium rabdosiin isolated from *Alkanna sfikasiana* Tan, Vold and Strid. *S Afr J Bot*, 2019, 120, 157-162.
2. Ganos, C.; Aligiannis, N.; Chinou, I.; Naziris, N.; Chountoulesi, M.; Mroczek, T.; Graikou, K. *Rindera graeca* (Boraginaceae) phytochemical profile and biological activities. *Molecules*, 2020, *25*, 3625 doi:10.3390/molecules25163625
3. Jeziorek, M., Damianakos, H., Kawiak, A., Laudy A.E., Zakrzewska, K., Sykłowska-Baranek, K., Chinou, I., Pietrosiuk, A. Bioactive rinderol and cynoglosol isolated from *Cynoglossum columnae* Ten. *in vitro* root culture. *Ind. Crops Prod*. 2019, *137*, 446-452. doi: 10.1016/j.indcrop.2019.04.046
4. Damianakos, H.; Jeziorek, M.; Sykłowska-Baranek, K.; Buchwald, W.; Pietrosiuk, A.; Chinou, I. Pyrrolizidine alkaloids from *Cynoglossum columnae* Ten. (Boraginaceae). *Phytochemistry Letters* 2016, *15*, 234–237, doi:10.1016/j.phytol.2016.02.005

Thin Layer Chromatography (cellulose type)

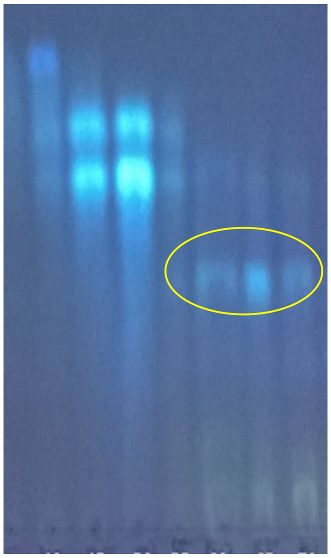

Rosmarinic acid (in yellow circle)

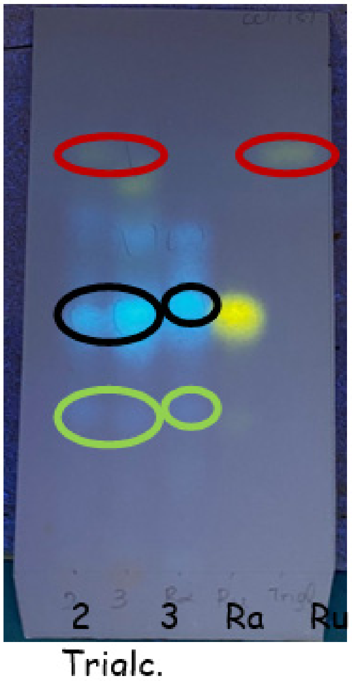

2=Shoots, 3=roots, Ra=Rosmarinic acid (black) and Lithospermic acid (green), Ru=rutin, Triglc= Quercetin 3-rutinoside-7-rhamnoside

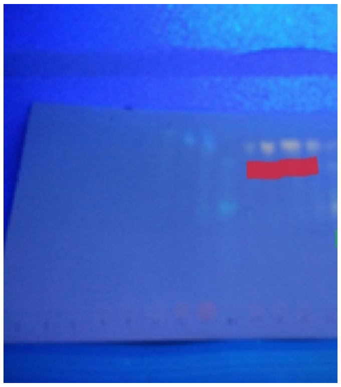

Quercetin 3-rutinoside-7-rhamnoside (red line)

Thin Layer Chromatography (silica type)

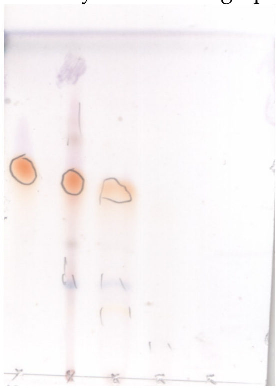

Rinderol (CH_2Cl_2/MeOH 99:1)
